# Exposure of primary osteoblasts to combined magnetic and electric fields induced spatiotemporal endochondral ossification characteristic gene- and protein expression profiles

**DOI:** 10.1186/s40634-022-00477-9

**Published:** 2022-05-02

**Authors:** Klaus H. Dittmann, Claus Mayer, Heribert Stephan, Christin Mieth, Michael Bonin, Beat Lechmann, H. Peter Rodemann

**Affiliations:** 1 Division of Radiation Biology, Dept of Radiation Oncology, Medical Faculty, Eberhard Karls University Tübingen, Roentgenweg 11, 72076 Tuebingen, Germany; 2 Neue Magnetodyn GmbH, Hermann-Oberth-Str. 9, 85640 Putzbrunn, Germany; 3 IMGM Laboratories GmbH, 10x Genomics B.V., Leiden, The Netherlands; 4 DePuySynthes, Synthes GmbH, Luzernstrasse 21, 4528 Zuchwil, Switzerland

**Keywords:** Primary human osteoblasts, Electromagnetic field, Wnt-signaling/TGFß-signaling

## Abstract

**Purpose:**

Molecular processes in primary osteoblasts were analyzed in response to magnetic and electric field exposure to examine its potential impact on bone healing.

**Methods:**

Primary osteoblasts were exposed to a combination of a magnetic field and an additional electric field (EFMF) (20 Hz, 700 mV, 5 mT, continuous sinusoids) in vitro. mRNA- and protein-expressions were assessed during a time interval of 21 days and compared with expression data obtained from control osteoblasts.

**Results:**

We observed an autonomous osteoblast differentiation process in vitro under the chosen cultivation conditions. The initial proliferative phase was characterized by a constitutively high mRNA expression of extracellular matrix proteins. Concurrent EFMF exposure resulted in significanly increased cell proliferation (fold change: 1.25) and reduced mRNA-expressions of matrix components (0.5–0.75). The following reorganization of the extracellular matrix is prerequisite for matrix mineralization and is characterised by increased Ca^2+^ deposition (1.44). On molecular level EFMF exposure led to a significant decreased thrombospondin 1 (THBS1) mRNA- (0.81) and protein- (0.54) expression, which in turn reduced the TGFß1-dependent mRNA- (0.68) and protein- (0.5) expression of transforming growth factor beta induced (ßIG-H3) significantly, an inhibitor of endochondral ossification. Consequently, EFMF exposure stimulated the expression of genes characteristic for endochondral ossification, such as collagen type 10, A1 (1.50), osteopontin (1.50) and acellular communication network factor 3 (NOV) (1.45).

**Conclusions:**

In vitro exposure of osteoblasts to EFMF supports cell differentiation and induces gene- and protein-expression patterns characteristic for endochondral ossification during bone fracture healing in vivo.

**Supplementary Information:**

The online version contains supplementary material available at 10.1186/s40634-022-00477-9.

## Background

In general, bone formation in vertebrates is performed by two processes: intramembranous and endochondral ossification [39]. Flat bones, which are found in the skull and mandible, are formed by intramembranous ossification, whereas most other bones are produced by endochondral ossification arising from a cartilaginous template [16, 34]. Bone fracture healing is achieved by endochondral ossification. On molecular and cellular levels, bone fracture healing can be broken down into different stages. Mesenchymal cells in the fracture area undergo hypertrophy, differentiate and simultaneously secrete extracellular matrix (osteoid) mainly consisting of collagen type I. Chondroclasts remove the cartilage matrix, and differentiating osteoblasts use the remnants of cartilage matrix as scaffolds for the deposition of bone matrix [9]. The initial step in osteogenesis is the differentiation of MSCs to osteoprogenitor cells, which is driven by wingless-type mouse mammary tumor virus (MMTV) integration site family members (WNTs) and bone morphogenetic proteins (BMPs) [58]. In this context, the expression of alkaline phosphatase (ALPL) is induced concomitantly, halting the proliferation and activating the differentiation of osteoprogenitor cells into pre-osteoblasts and mature osteoblasts, which accumulate and mineralize the extracellular matrix [2, 4, 24]. Finally, osteoblasts transit to osteocytes with reduced alkaline phosphatase expression and increased osteocalcin (SPP1) expression [7]. Osteocytes are enriched in proteins that confer resistance to hypoxia, which is essential for their embedded location and restricted oxygen supply [11]. Clinical studies based on the application of an electromagnetic field (EFMF) stimulation (combined stimulation with an alternating external magnetic field (5 mT), that induces an electric potential of 700 mV in an implanted transducer) have been successful in the treatment of wound and bone fracture healing as well as nonunions [3, 30, 43] fractures of the femoral neck, avascular necrosis of the femoral head and pain reduction [27]. As investigated in in vitro cell culture studies at the level of human fibroblast cultures, exposure to electromagnetic fields has the potential to stimulate terminal differentiation of fibroblasts into functioning, highly collagen producing fibrocytes predominantly through modulation of Ca^2+^-influx and activation of protein kinase A [31, 40]. Similarly, studies using in vitro cultured primary osteoblasts of different species also revealed that exposure to magnetic fields (MF) can stimulate protein kinase A (PKA) activity [14, 51]. Moreover, through the use of mesenchymal stem cells, magnetic field exposure has been demonstrated to stimulate osteogenic differentiation through activation of the PKA and ERK1/2 pathways [57] as well as chondrogenic differentiation [32, 35, 42, 52]. Similar effects have been observed for mesenchymal stem cells exposed in vitro to an alternating electric current of 5–40 μA at a frequency between 5 and 10 Hz (EF) [10, 42, 55]. Zhou described a PKA-driven optimal effect of electromagnetic fields with 50 Hz and 1.8 mT on osteogenic differentiation and mineralization in an animal model [60].

Considering the outcomes of previous studies, in the present study, we investigated the potential effect of combined specified electric and magnetic field (EFMF) exposure on primary human osteoblasts to modify cellular and molecular biological pathways in vitro and the parameters that may support and stimulate the biological processes of bone fracture healing in vivo.

## Methods

### In vitro cell cultures

*Human primary osteoblasts (hOBs)* designation: A12720 (lot. 423Z010.2 and 422Z047.2) cells were obtained from two male donors (age 58 and 43 y, femoral trabecular bone tissue from the knee or hip joint region) and were purchased from *Promocell* (Heidelberg, Germany). Data obtained from the two lots of primary osteoblasts were pooled. These cells stained positive for alkaline phosphatase and were cultivated using Osteoblast Growth Medium *(PromoCell)*.

### EFMF exposure system and conditions

Passage 5 cultures of primary osteoblasts were plated into culture vessels (24-well plates depending on the test parameters) and exposed to the following control and test conditions:

The external electromagnetic field was generated with specially designed solenoid coils (FA-WP24-K, Neue Magnetodyn, Putzbrunn, Germany) combined with a frequency generator (M70, Neue Magnetodyn, Munich, Germany). The solenoid was horizontally positioned within an incubator. To prevent heating of the solenoid and to ensure a stable temperature of 37 ± 0.1 °C, the coil was surrounded by a cooling enclosure that was connected to a water bath. The temperature of the water bath was controlled by a thermostat combined with a cooling unit (Haake, Verden, Germany). The electric component was generated by electromagnetic induction of a transducer (secondary inductivity) by the primary magnetic field. The resulting voltage was 700 mV_RMS_, 20 Hz. This voltage was applied to the cells by specially designed well-plates with interdigitated electrodes made of fine gold on the ground of the well-plates (Fig. S2 and S3).

The EFMF treatment was a combined exposure to an electric field (EF, 20 Hz, 700 mV) and a magnetic field (MF, 20 Hz, 5 mT), both continuous sinusoids, 4 × 45 min / day, i.e., every 6 h.

These are the same parameters as used in clinical applications of a Magnetodyn System [3, 27, 30]. Osteoblasts were seeded and grown for three days before first exposure. Control cells were cultured in a parallel incubator in solenoid coils and in the specially designed well-plates with gold electrodes, but without field exposure for 0, 3, 7, 14, and 21 days under identical physiological environmental parameters (temperature, CO_2_ concentration, humidity). At each time point, exposed and control cells were prepared for cell and molecular biology analyses.

### Alizarin staining

hOBs were seeded in 24-well plates at various densities (3125–50,000 cells/well (2 cm^2^)) depending on the incubation time (0–21 days) to avoid confluency. After exposure to different fields, cells were fixed at days 0, 3, 7, 14, and 21 by incubation with 70% ethanol. After washing with H_2_O, the cells were stained with Alizarin red solution (0.5%) (Millipore) for 45 min in the dark. Culture wells were washed three times with H_2_O and dried. Since the bottom of the exposure wells was opaque, we resolved cell-bound stain by incubation with 70% ethanol for 2 h with shaking. Alizarin red was quantified with an ELISA reader at 405 nm. ODs were normalized to the protein amount detected in the wells by protein assay (Bio-Rad).

### Total RNA extraction for genome-wide expression analyses

hOBs were seeded in 24-well plates at various densities (3125–50,000 cells/well) depending on the incubation time (0–21 days) with or without EFMF exposure. After exposure, cells were harvested at days 0, 3, 7, 14 and 21 and lysed for isolation of total RNA using the Qiagen miRNeasy Micro Kit. For each control and test condition and time points, three technical and three biological replicates were performed and extracted total RNA was delivered to the project partner IMGM Laboratories GmbH for mRNA expression profiling. mRNA libraries were generated using Illumina TruSeq® Stranded mRNA technology and the NextSeq® 500 next-generation sequencing system (Illumina). Generated reads were mapped against the human reference genome (GRCh38.p7) using the CLC Genomics Workbench (Qiagen). Differential gene expression between groups (stimulated vs. control) was calculated using CLC for each time point and biological replicate separately.

### Protein expression analyses

Cells were seeded at the specified densities per well of 24-well plates (hOBs 3125–50,000 cells/well), depending on the incubation time (0–21 days). After exposure, the cells were harvested at days 0, 3, 7, 14, and 21 and lysed for protein extraction. At different time points after exposure to control and test conditions cell lysates were analyzed by SDS-PAGE and Western blotting. Quantitative analysis of the specific intensities of total proteins was performed by applying the LICOR system Odyssey FC. Notably, the selection of marker proteins relevant for proliferation and differentiation, as well as ossification of human primary osteoblasts, as demonstrated in the *Results,* was based on the mRNA expression profiles resulting from the transcriptome analyses provided by the project partner IMGM Laboratories GmbH.

### Statistical tests

The data sets were tested for differences by means of the Kruskal–Wallis test. If there were significant differences, Mann–Whitney tests with Bonferroni adjustment were performed in pairs post hoc.

## Results

### Morphological and molecular characterization of primary osteoblasts during 21 day in vitro cultivation

**Fig. 1:**
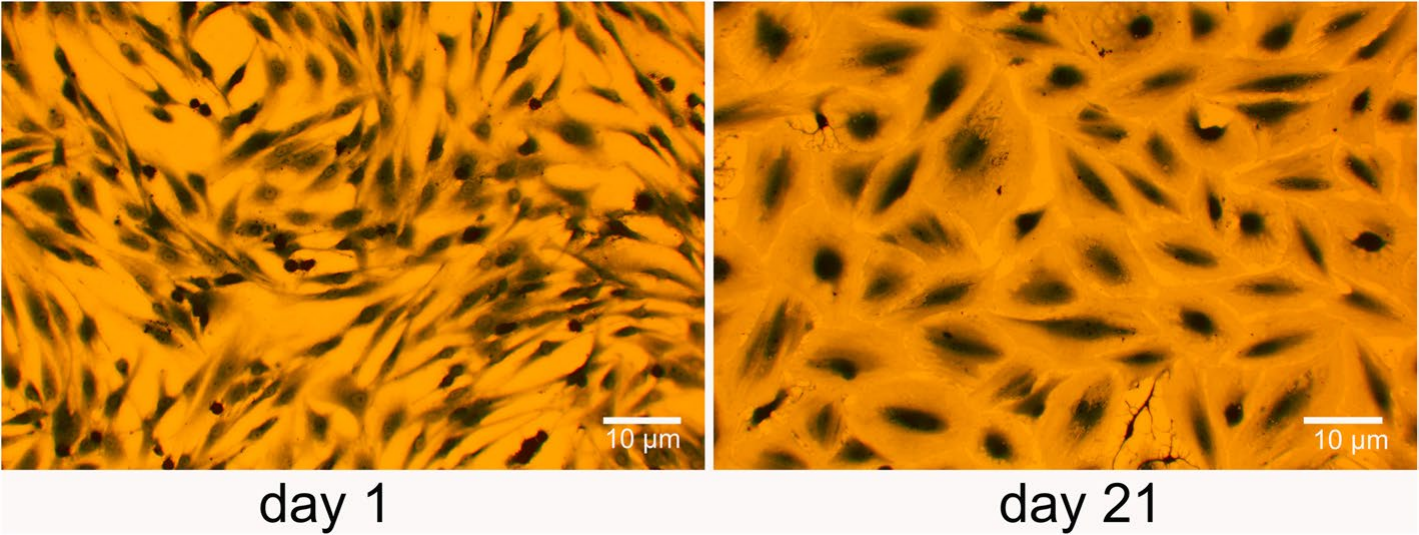
Phenotypic alterations of human primary osteoblasts during 21 day in vitro cultivation

To elucidate the molecular effect of an EFMF exposure on human osteoblasts, we performed an in vitro cultivation of human osteoblasts (PromoCell) for 21 days. These cells stained positive for alkaline phosphatase and were positive for induction of mineralization. Osteoblasts were cultivated with the specified growth medium according the instructions of “PromoCell”. During the cultivation over 21 days, we observed a distinct morphological alteration to cells with enlarged cytoplasms and nuclei (Fig. 1). This altered cell phenotype correlated with increased matrix mineralization (Fig. 5) and was characterized by high mRNA-expression for alkaline phosphatase (ALPL), cathepsin K (CTSK), cartilage oligomeric matrix protein (COMP), cadherin 11 (CDH11), fibronectin (FN1), osteopontin (SPP1), thrombospondin 1(THBS1), metallopeptidase inhibitor 1 (TIMP1) and continuous high expression of collagen type I (COL1A1) and Anexin 5 (ANXA5) (Table 1).

**Table 1:**
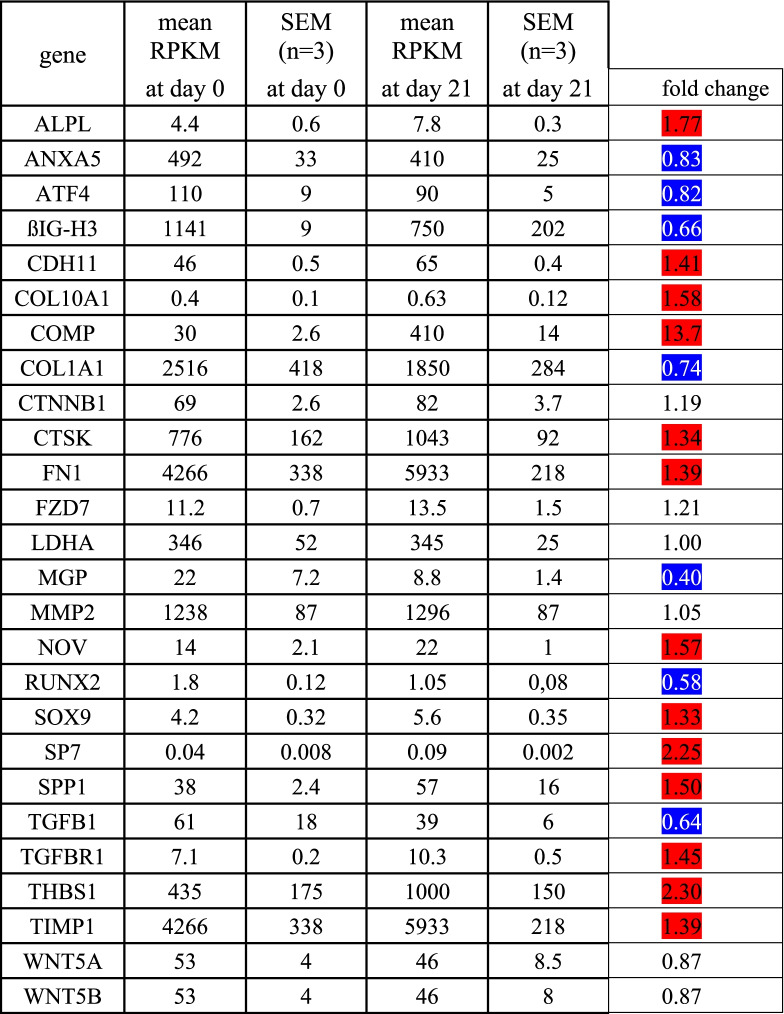
mRNA-expression of osteogenic genes in human osteoblasts after 0 and 21 days cultivation. Significant increase (*p* < 0.05) is given in red, significant reduction (*p* < 0.05) is given in blue. mRNA expression of osteogenic genes in human primary osteoblasts at day 0 and 21 of cultivation. Shown is the mean RPKM +/− SEM (reads per kilobase of transcript per million mapped reads) from 3 independent experiments

### EFMF induced proliferation of primary osteoblasts in vitro

To evaluate the molecular effects of combined exposure to EF and MF, we exposed human primary osteoblasts (hOBs) under standardized conditions to a combination of an electric (20 Hz, 700 mV) and a magnetic field (20 Hz, 5 mT) (EFMF).

**Fig. 2:**
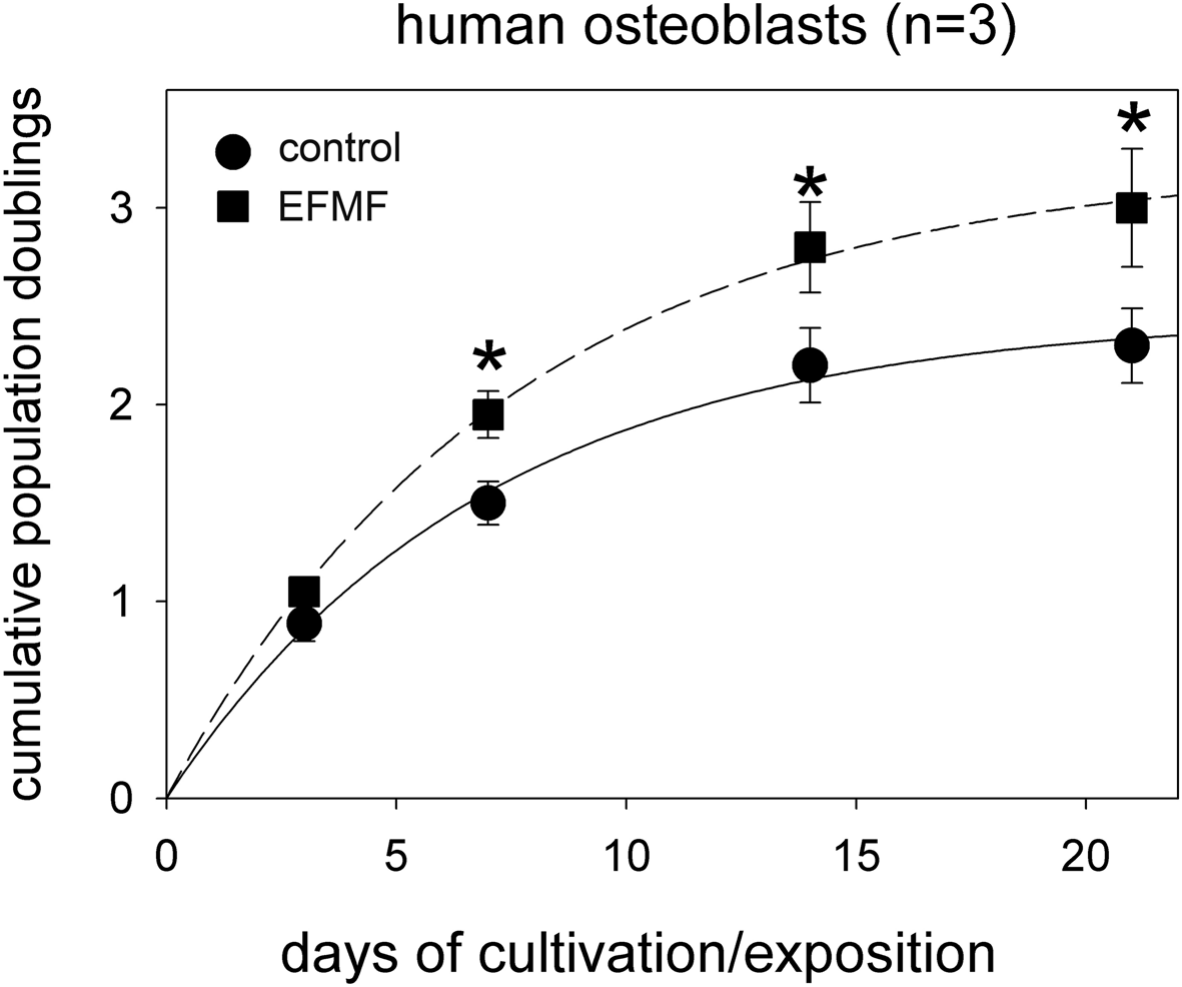
Effect of EFMF exposure on cell proliferation of hOBs. Cumulative population doublings in EFMF-treated hOBs over 21 days of exposure. * *p* < 0.05 as compared to control cells. Data are derived from 3 independent experiments

Figure 2 gives the cumulative population doublings of primary osteoblasts during the 21 days cultivation / exposure time. We observed cell proliferation up to day 14 followed by a stationary phase. During the proliferation phase exposure to EFMF increased the proliferation rate (*p* < 0.05) up to about 25%. To avoid inhibitory effects of contact inhibition, cells were seeded at a reduced number when cultivated for longer periods.

### EFMF induced alterations in the transcriptome

**Fig. 3:**
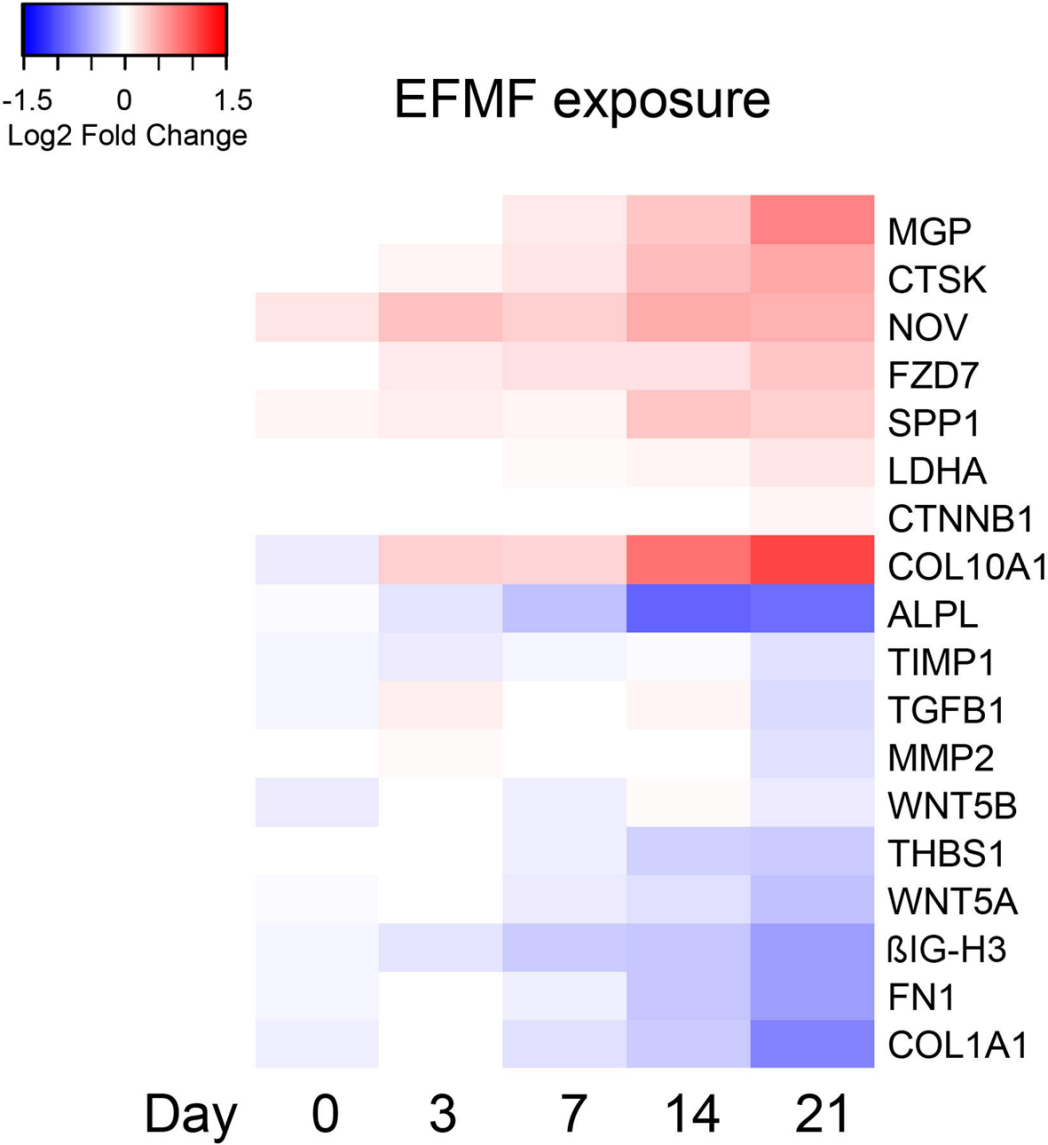
Differential expressed genes of human primary osteoblasts treated with EFMF vs. control. The heat map shows the mean log2-fold changes in expression of genes important for osteoblast differentiation from 3 independent experiments at time points 0, 3, 7, 14, and 21 days. (see also Fig. S1)

To elucidate the molecular processes triggered by the applied EFMF exposure, we quantified changes in the transcriptome of exposed versus control hOBs (Fig. 3, Fig. S1).

The observed proliferative effect of EFMF exposure was reflected by the expression of marker of proliferation Ki67 (MKI67) mRNA. Under EFMF exposure, increased expression was observed at day 3 (fold change: 1.32), which correlates with the observed increased proliferation (Fig. 2). However, MKI67 mRNA expression declined during the 21 day cultivation (0.39) (Fig. S1), which is in agreement with the reduced cell growth at later time points, as presented in Fig. 2. In control cells, cellular avian myelocytomatosis homolog (MYC) (0.47) and insulin like growth factor binding protein 3 (IGFBP3) (0.55) gene expression declined and cyclin dependendent kinase inhibitor 1A (CDKN1A) expression increased (1.64) during the 21 day cultivation period. EFMF exposure slightly decreased expression of MYC or CDKN1A (Fig. S1). To elucidate the molecular processes regulating the growth of primary osteoblasts and EFMF-induced proliferation, we investigated the expression of members of the WNT signaling pathways. Primary osteoblasts were characterized by high mRNA expression of Frizzled Class Receptor 7 (FZDZ) and of its ligands WNT5A and WNT5B (Table 1, mean RPKM)). However, in response to EFMF exposure, the mRNA expression of FZDZ (1.25) was further increased, whereas the expression of WNT5A (0.8) decreased (Fig. 3) during the 21-day cultivation**.**

### EFMF induced proteome alterations in primary osteoblasts

**Fig. 4:**
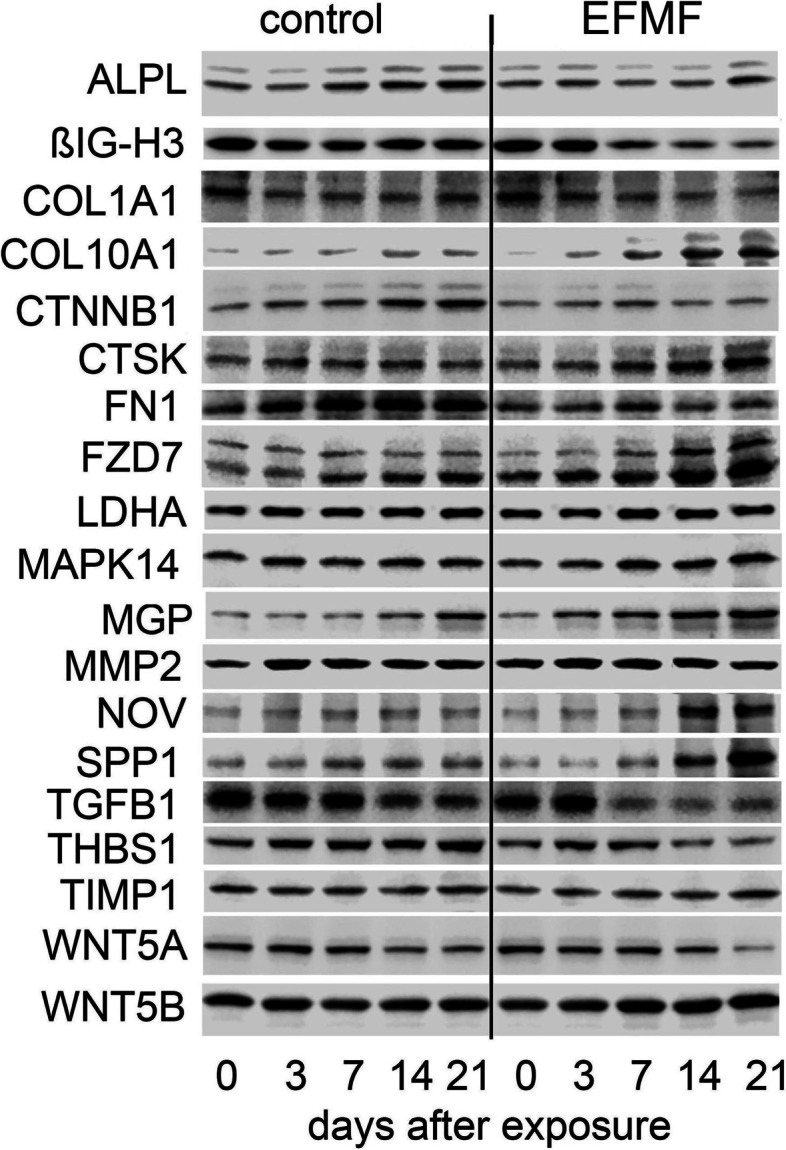
Time-dependent protein expression of selected proteins of hOBs in response to EFMF treatment. Relative protein expressions were corrected to that of ß-Actin in the individual blots. Densitometric quantification is presented in Table 2

In agreement with FZD7 mRNA expression (Fig. 3), we observed distinct FZD7 protein expression in primary osteoblasts, which increased during cultivation over 21 days (Fig. 4, Table 2). EFMF exposure for 21 days further elevated FZD7 protein expression (1.53) (Fig. 4, Table 2). Although the FZD7 ligand WNT5B was expressed at a high protein level in control cells, its expression was further increased upon EFMF exposure (1.35), whereas that of the FZD7 ligand WNT5A declined in response to EFMF (0.51) (Fig. 4, Table 2). Because of WNT signaling, primary osteoblasts expressed increasing amounts of CTNNB1 protein during in vitro cultivation (1.2) (Table 1). However, EFMF exposure reduced CTNNB1 protein expression (0.3), as observed for the WNT5A protein (Fig. 4, Table 2). In contrast to mRNA expression of the WNT ligand WNT5B (0.83) (Fig. 3), protein increased in response to EFMF treatment (1.46) (Fig. 4, Table 2), which may be indicative of induction of a cell differentiation process. In agreement with the idea of a differentiation process, we observed that osteoblasts used in these experiments (day 0) were positive for alkaline phosphatase (ALPL) and MAPK 14 expression, which was confirmed at protein expression levels (Fig. 4). After seeding, within the 21-day culture period, the expression of ALPL mRNA in the control osteoblasts increased continuously from day 0 to day 21 (1.77) (Table 1). In EFMF-exposed osteoblasts, this increase was not detectable and was given as a reduced relative expression in Fig. 3, which presents the differential ALPL mRNA expression (0.57). In agreement with this observation on mRNA level, the ALPL protein expression increased clearly in control cells (1.6) compared to the EFMF-exposed cells during the culture period of 21 days (0.7) (Fig. 4, Table 2).

**Table 2:**
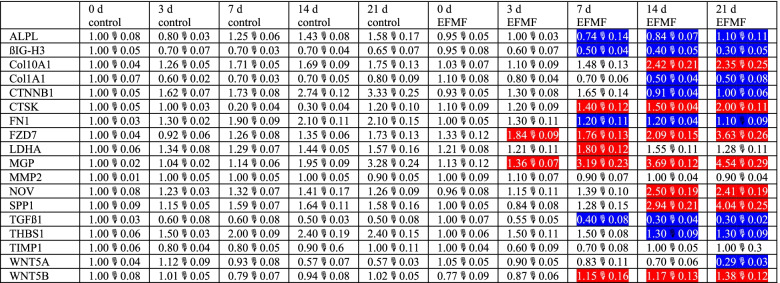
The fold protein expression of Fig. 4 is given below as mean of 3 independent experiments (± SEM). Significant increase induced by EFMF exposure is given in red (*p* < 0.05), significant reduction (*p* < 0.05) is given in blue

### EFMF induced mineralization in primary osteoblasts

**Fig. 5:**
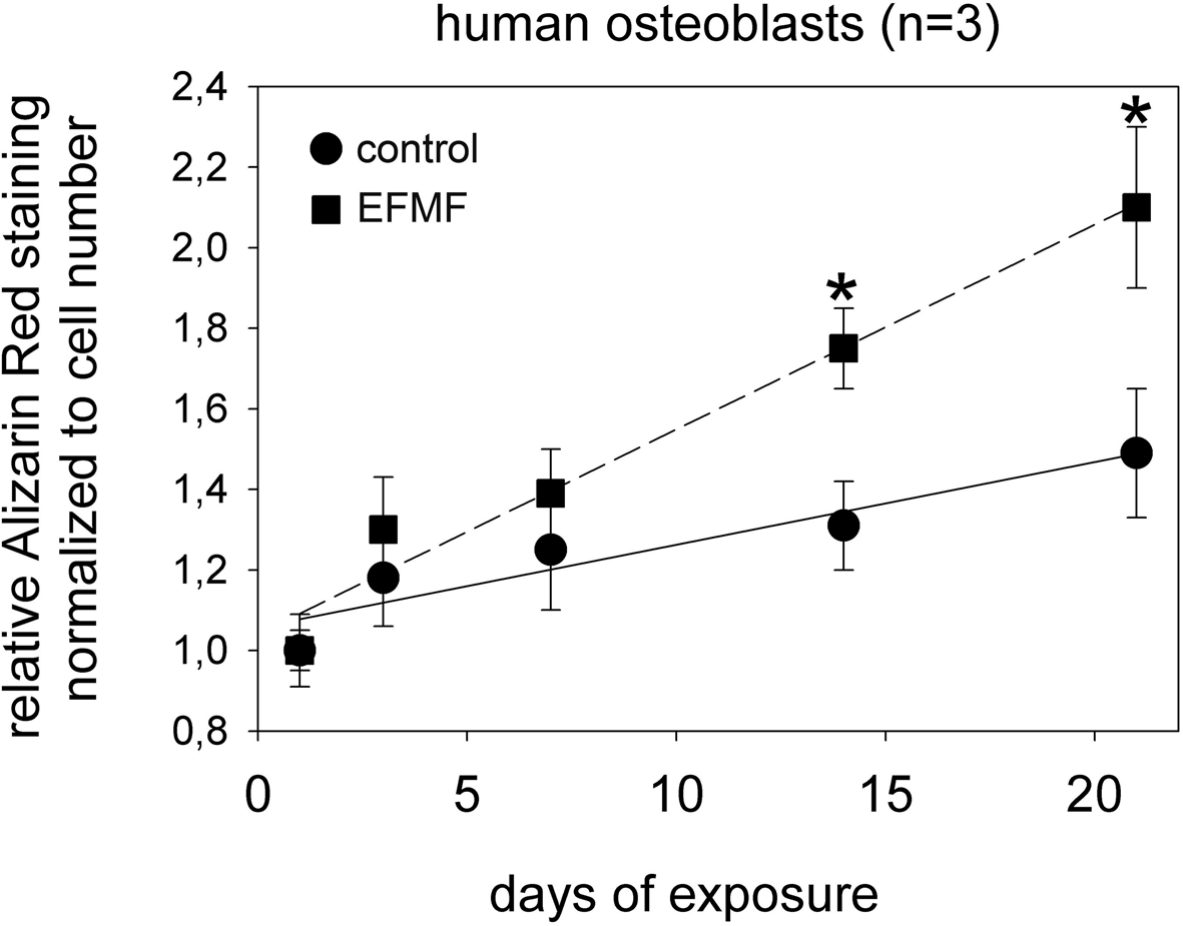
Alizarin red staining of hOBs after exposure to EFMF. Staining was quantified with an ELISA reader at 405 nm, and the results were normalized to the cell number detected in the exposure wells. *n* = 3, * = *p* < 0.05 compared to control cells (normalized on cell counts)

To elucidate the functional relevance of the phenotypical alterations observed during the 21 day cultivation period with or without EFMF exposure, we performed an Alizarin Ca^2+^ staining. Cultivation of primary osteoblasts over 21 days was associated with a continuous small increase in Ca^2+^ deposition (1.11). In agreement with this finding, a high expression of Ca^2+^-associated structural genes such as Catherin11 (1.40)(CDH11) and cartilage oligomeric matrix protein (COMP) (1.50) was observed. Exposure to EFMF resulted in further and significantly increased cellular Ca^2+^ deposition (1.44) (Fig. 5). The observed changes in phenotype and the increased Ca^2+^ deposition in EFMF-exposed osteoblasts indicated an induced differentiation process of the applied hOBs. To characterize the associated functional alterations, we summarized the protein expression (Fig. 4) of essential genes involved in osteoblast differentiation and function that responded to EFMF.

### EFMF induced the expression of endochondral ossification markers in primary osteoblasts

Upon EFMF exposure, significantly induced expression of osteogenic genes involved in endochondral ossification, such as collagen type X, alpha chain (COL10A1)(1.50), matrix Gla protein (MGP)(1.55), osteopontin (SPP1)(1.50) and cellular communication network factor 3 (NOV)(1.45), was observed (Fig. 3). Most interestingly, after 7 days of EFMF exposure, the expression of lactate dehydrogenase (LDHA)(1.39), which is a characteristic marker of aerobe glycolysis, was increased (Figs. 3 and 4). Similarly, the induction of aerobe glycolysis was confirmed by the mRNA expression of hypoxia inducible factor 1 subunit alpha (HIF1A)(1.3) and vascular endothelial growth factor B (VEGFB)(1.5) (Fig. S1.).

### EFMF induced the reduced expression of extracellular matrix components in primary osteoblasts

On the other hand, 21 days of EFMF exposure resulted in reduced mRNA expression of extracellular matrix components such as COL1A1 (0.7), COL1A2 (0.75), COL5A1 (0.6) and FN1 (0.6) (Fig. S1); these compounds are usually expressed in high quantities by primary osteoblasts (Table 1). Within the 21 day period of EFMF exposure, the extracellular matrix-degrading enzyme MMP2 and its inhibitor TIMP1 were detected at high protein levels (Fig. 4). These data indicate high constitutive matrix turnover.

Notably, the cysteine proteinase cathepsin K (CTSK), an osteoclast-specific enzyme, involved in bone remodeling and resorption, was strongly expressed under control conditions at day 21 (Table 1). Exposure to EFMF for 21 days resulted in further stimulated mRNA and protein expression of CTSK (1.6) (Figs. 3 and 4).

### EFMF induced osteogenic signaling in primary osteoblasts

To elucidate potential molecular regulation processes triggering the expression of structural proteins involved in bone formation regulation, we checked the expression profile of known regulators, such as RUNX2, ATF4, SP7, and SOX9, as well as components of TGFβ signaling. These analyses revealed low expression of RUNX2 (Table. 1). The expression further decreased during the 21-day cultivation time independent of EFMF exposure (Fig. S1). This decrease in RUNX2-expression (0.82) is in accordance with proceeding bone maturation [8]. ATF4 was highly expressed in primary osteoblasts (Table 1) but decreased over 21 days of cultivation under both control and exposure conditions sightly (0.75) (Fig. S1). SP7 (Osterix), also an important factor for osteoblast differentiation, was expressed only at very low levels (Table 1). In contrast primary osteoblasts presented high levels of SOX9 expression (Table 1), which was markedly increased upon EFMF exposure (1.50) (Fig. S1).

### EFMF modulated TGFß signaling in primary osteoblasts

Several regulators and members of the TGFβ signaling cascade were expressed constitutively in primary osteoblasts on high mRNA levels (Table 1) and EFMF exposure did not result in an altered expression*.* This is true for the TGFß1-receptor TGFBR1 (0.93) and also for its ligand TGFß1 (TGFB1) (0.9). However, on protein level a significant reduction of active TGFB1 protein was observed (0.6) in response to EFMF exposure (Fig. 4, Table 2). This observation suggests, that the amount of active TGFB1 protein is regulated in response to EFMF exposure. Formation of active TGFB1 from its latent form is stimulated by Thrombospondin 1 (THBS1) [18]. Indeed, THBS1 mRNA (0.81) (Fig. 3) and protein (0.54) (Fig. 4, Table 2) were signifcantly reduced in response to EFMF exposure, which correlates with reduced TGFB1 protein (Fig. 4, Table 2). Consequently, TGFB1 dependent gene expressions should be reduced. Indeed, we observed reduced mRNA expression of TGFB1 dependent ßIG-H3 (0.68) (Fig. 3) (alias TGFBI, TGFß-induced) in response to EFMF exposure. In addition, also ßIG-H3-protein expression was reduced clearly (0.50) (Fig. 4, Table 2). It is noteworthy, that ßIG-H3 was very highly expressed in control osteoblasts (Table 1) and acts as an inhibitor of osteoblast differentiation and endochondral ossification [6].

In summary, EFMF exposure stimulated mRNA and protein expression of members of the WNT-signaling and blocked TGFβ-signaling pathway at the end of osteoblastic differentiation process to enable endochondral ossification.

## Discussion

Osteoblast differentiation in vitro and in vivo proceeds in three phases: cell proliferation, matrix maturation and matrix mineralization [48]. This differentiation process can be increased by growing cells to confluence and by addition of specific osteogenic factors [20].

Human primary osteoblasts were cultured in growth medium (*PromoCell)* for 21 days. To elucidate the molecular effect of a magnetic field exposure, we treated primary osteoblasts by a combination of a magnetic field and an intensified electric field (EFMF) (20 Hz, 5 mT, 700 mV, continuous sinusoids) and assessed mRNA and protein expressions over 21 days.

Data reported herein indicate that 21-day EFMF exposure of human primary osteoblasts in vitro is associated with expression of proteins essential for endochondral ossification, which is necessary for the biological signaling cascades critical to bone fracture healing. Exposure of osteoblasts to EFMF resulted in an increase in the total cell numbers after 21 days of EFMF exposure, reflecting stimulated cell proliferation. Comparable proliferative effects on osteoblasts in response to magnetic field exposure alone have been reported by various other researchers [9, 14, 15, 22, 38, 56].

In contrast to control osteoblasts, EFMF-exposed osteoblasts exhibited a significantly stimulated proliferation rate between days 7 and 14. This differential growth kinetic of control and EFMF-exposed osteoblasts was mirrored by the mRNA expression level of the proliferation marker MKI67, which showed a significant increase at day 3. The observed subsequent decline in cell proliferation of EFMF-exposed cells up to day 21 was reflected precisely by the stimulated expression of the cyclin kinase inhibitor CDKN1A, known as a negative cell growth control factor [58].

The phenotypic alteration observed during cultivation time (cytoplasm and nucleus enlargement) is in accordance with the observation of Kim in spheroid cultures [21], indicating that during the differentiation process osteoblasts condensate to osteocytes. In agreement with that, we observed in our cultures after 21 day cultivation an increased expression of osteocyte markers, e.g. SPP1, HIF1A, LDHA, VEGFB and a reduced expression of alkaline phosphatase (ALPL) upon exposure to EFMF, which suggests an induced osteoblasts transition to osteocytes.

In general, and independent of the exposure conditions, the initial gene expression profile of the osteoblast preparation applied was characterized by high expression levels of extracellular matrix proteins such as COL1A1, COL1A2, COL5A1, CDH11 (cadherin 11), FN1 (fibronectin), ANXA5 (annexin A5), THBS1 (thrombospondin 1), COMP (cartilage oligomeric matrix protein), and DCN (decorin). These observations were in accordance with the reports by Kozloff (2015) and Kim (2008) [1, 16]. For both exposed and control cells, the mRNA expression levels of ANXA5, COL1A1, COL1A2, COL5A1 and DCN declined over the 21-day cultivation period, whereas the mRNA expression levels of COL15A, FN1, THBS, CDH11 and COMP mRNAs increased. These differential effects most likely indicate a reorganization process of the extracellular matrix during the applied cultivation period. Similarly, within the 21-day cultivation period, the mRNA expression of matrix metalloproteinase MMP2 decreased slightly, but persisted on a high level. In contrast, both the mRNA expression of the proteinase CTSK (Cathepsin K) and that of ALPL (alkaline phosphatase) increased continuously over the 21-day culture period. This finding is of particular interest, as ALPL expression, which itself is dependent on TGFß [23], is an important regulator of cartilage mineralization [24]. Mineralization is an essential feature of bone remodeling during fracture healing [44]. Moreover, EFMF exposure of primary osteoblasts resulted in decreased expression of ALPL and various collagen- and collagen-related genes, but stimulated the expression of SPP1 and COL10A1. The results indicate that EFMF exposure is able to accelerate osteoblastic differentiation and to induce osteoblasts switching into a phenotype prerequisite for endochondral ossification [13, 25, 46]. Therefore, in agreement with the observed increase in COL10A1 and SPP1 mRNA- and protein-expression, we detected increased mineralization of ECM upon EFMF exposure.

The biological impact of EFMF-induced molecular alterations mediating endochondral ossification processes in cultured osteoblasts observed in the present study was demonstrated under identical technical conditions of EFMF exposure regimens in vivo in tibial osteotomy models in sheep performed by Darwiche [12]. In the study by Darwiche, the EFMF treatment was able to stimulate an accelerated and enhanced bone fracture healing process, which resulted in an improved bone structure and callus morphology as well as superior biomechanical properties.

To elucidate the underlying regulatory molecular processes stimulated by EFMF exposure, we identified Wnt signaling [37] as responding molecular switch. As demonstrated herein, exposure of osteoblasts to EFMF induced a distinct increase in the expression of the Wnt receptor FZD7. As reported, the corresponding ligands WNT5A and WNT5B are constitutively highly expressed in osteoblasts [8, 45] and can act in an autocrine manner on FZD7. The potential of WNT5B to promote cell proliferation and migration described by Zhang [59] may explain the enhanced proliferative effect of EFMF exposure on the osteoblasts observed in our study. WNT5A, known as the “typical” noncanonical WNT ligand, is able to repress and activate WNT-dependent signaling both in vitro and in vivo [36, 53]. It is known, that stimulated expression of MAPK14 is necessary for Wnt-dependent stimulation of proliferation [54]. As shown herein, the requirement of stimulated MAPK14 expression to mediate osteoblast proliferation was confirmed with EFMF-exposed osteoblasts. Moreover, and important in this context, it has clearly been shown [41], that stimulated MAPK14 expression can also promote osteoblast differentiation and trigger differentiation of osteoprogenitors to osteoblasts and osteocytes. In agreement with Shao [45], we observed an EFMF-induced Wnt signaling, which leads to the upregulation of catenin ß1 (CTNNB1) a regulatory component of bone formation [19, 26].

As presented herein, EFMF-exposed osteoblasts express all markers of osteoblastic differentiation including osteocalcin. In agreement with our results, and as described by Luttrell [33], Wnt/ß-catenin signaling is a prominent player during early osteoblast differentiation. During proceeding differentiation, Wnt expression is reduced to allow further maturation controlled by other signaling inducers, e.g., TGFB/BMP [8]. Indeed, we observed constitutive expression of TGFB1, SMADs and BMPs. However, towards the end of the 21-day exposure period, the expression of these signal transducers decreased, except that of TGFB receptor 1 and 2. However, most importantly, the expression of TGFB1, known as a strong inducer of ßIG-H3 (TGFBI) transcription [29, 47, 50], was significantly decreased in osteoblasts exposed to EFMF. In the context of our study, it is most interesting that ßIG-H3 has been described as a negative regulator of osteoblast differentiation and endochondral ossification before [6, 16, 24]; these authors state that decreased expression of ßIG-H3 is associated with increased expression of collagen type X, a marker of endochondral ossification. In the context of our data, the previous results imply that EFMF exposure of primary osteoblasts is able to induce the endochondral ossification process by downregulating ßIG-H3 [5]. However, in our study, TGFB1 mRNA expression was not affected by EFMF exposure directly. However, we observed a clear repression of trombospondin 1-mRNA (THBS1) and protein expression upon EFMF exposure. Since THBS1 protein stimulates TGFB1 activity by increased formation of active TGFB1 from its latent form [18], reduced THBS1 expression will reduce formation of active TGFB1 upon EFMF exposure and may explain the decreased ßIG-H3 inhibitor mRNA and protein abundance. Low ßIG-H3 in response to EFMF exposure may support the process of endochondral ossification.

Interestingly, EFMF exposure induced distinct NOV (CCN3) expression, which is a strong positive regulator of chondrogenic differentiation and is able to induce collagen type X (Col10A) expression, as described by Lafont [28].

**Fig. 6:**
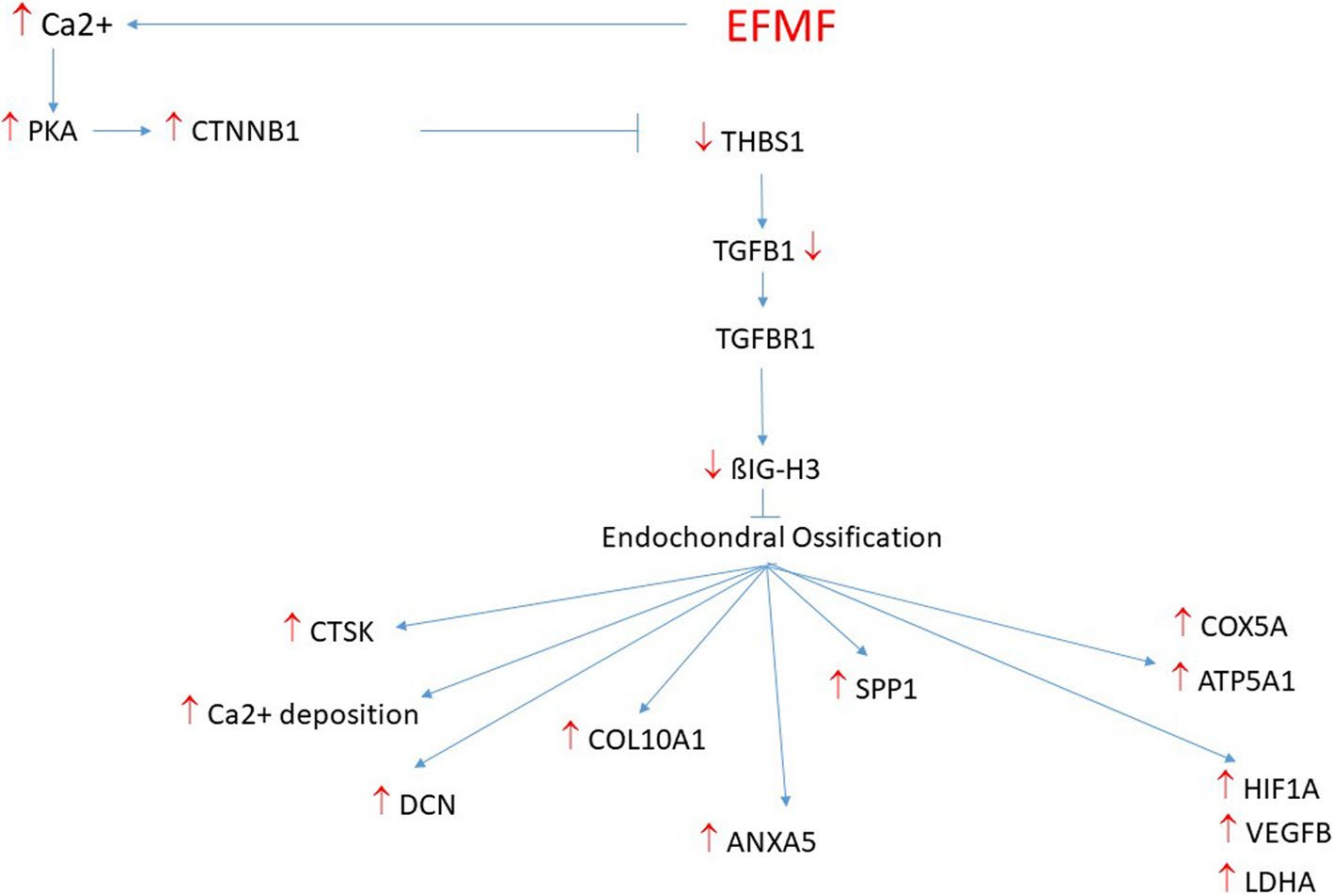
Suggested signalling pathway for EFMF-induced endochondral ossification. Primary osteoblasts are characterized by high constitutive wnt- and TGFß1- (TGFB1) signaling. The latent TGFB1 converts to its active form under control of trombospondin 1 (THBS1). Active TGFB1 binds to its receptor TGFBR1 and regulates expression of ßIG-H3, which is a negative regulator of endochondral ossification. EFMF exposure increases calcium channel expression and results in Ca^2+^ influx. This increased Ca^2+^ levels activate protein kinase A (PKA). Activated PKA can phosphorylate catenin beta 1(CTNNB1), which prevents its degradation. The stabilized transcription factor CTNNB1 blocks THBS1-expression and subsequently TGFB1 activation. Reduced TGFB1 activation and EFMF induced reduced TGFB1 expression decreased TGFB1-signalling. This results in reduced expression of the inhibitor ßIG-H3 and stimulation of endochondral ossification. Endochondral ossification linked differentiation is characterized by increased expression of collagen type X (COL10A1), decorin (DCN), annexin A5 (ANXA5), osteopontin (SPP1), cathepsin K (CTSK), hypoxia inducible factor 1 subunit α (HIF1A), vascular entothelial growth factor B (VEGFB), lactate dehydrogenase A (LDHA), cytochrome C oxidase subunit 5A (COX5A), ATP synthase F1 subunit alpha (ATP5A1). and increased Ca-deposition (Fig. 5)

As discussed above, Wnt signaling plays an important role in regulation of cell proliferation as well as of cell differentiation. Currently, it is not clear how EFMF exposure is able to activate initial Wnt signaling. A plausible explanation is given by Rodemann [31, 40, 51], who observed increased Ca^2+^ fluxes in response to MF exposure. Indeed, MF at low frequency can increase calcium channel expression and explain increased Ca^2+^ influx [49]. This increased Ca^2+^ levels result in protein kinase A (PKA) activation [51]. PKA can phosphorylate catenin ß1 (CTNNB1), which prevents its degradation [17] and blocks THBS1 expression [18], resulting in reduced TGFB1-signalling and downregulation of ßIG-H3 stimulating endochondral ossification (Fig. 6). These previously described cellular mechanisms and principles of the potential mode of action of electromagnetic field exposure are supported by a recent study by Zhou 2021, who applied inductively coupled electromagnetic fields (IC) in a rat osteoblast system. These authors showed, also that exposure to sinusoidal electromagnetic fields promotes osteogenic differentiation and bone formation through activation of protein kinase A [60]. In this context it is important to mention, that Rodemann applied inductively coupled electromagnetic fields (IC), which are an integrative part of the combination of alternating magnetic and electric fields (CS), we used in our experiments and were also used by Darwiche [12] in a sucessful animal study. To make clear which part of the combined exposure is responsible for the molecular effects observed herein, we plan to perform a series of experiments, where we will expose osteoblasts either to magnetic, electric or combined fields.

## Conclusions

**Fig. 7:**
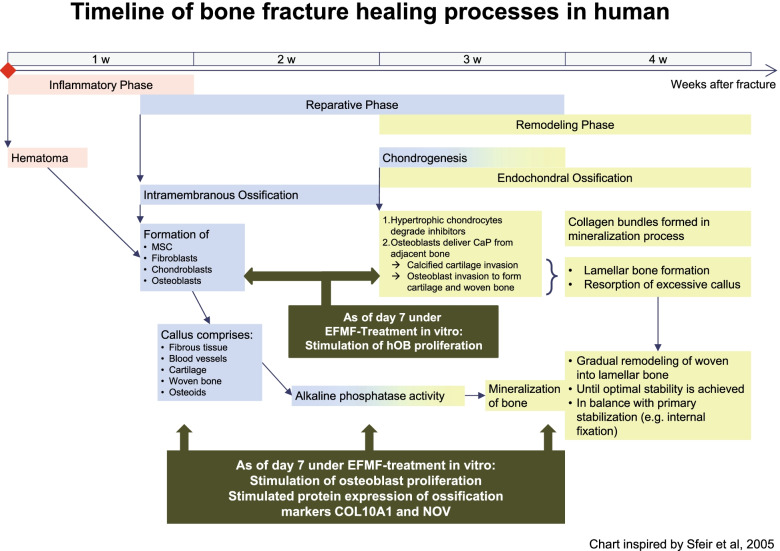
Cellular and physiological processes relevant for bone fracture repair. The expression of osteogenic proteins such as COL10A1 and NOV is stimulated after day 7, supporting alkaline phosphatase activity. hOB is stimulated when the endochondral ossification phase starts, and cartilage is remodeled into woven bone. The results are illustrated as a graphic representation of the bone healing process according to Sfeir [44]. However, the illustration purposefully focuses on the first four weeks after fracture, although the bone remodeling process continues

We observed an EFMF-induced signaling in osteoblasts in vitro activating an intrinsic, spatiotemporal gene regulatory network, which mediated the expression of proteins characteristic for endochondral ossification. Based on the data discussed, Fig. 6 presents the proposed model of EFMF-induced signaling in osteoblasts stimulating endochondral ossification that is relevant for improved bone fracture healing in vivo, as observed by Darwiche [12]. Taken together, the in vitro results presented herein fit perfectly mechanistically to the temporal, tissue-specific, biochemical processes relevant for bone fracture healing in vivo [44] (Fig. 7).

## Supplementary Information

**Additional file 1: Fig. S1.** Differential expressed genes of human primary osteoblasts treated with EFMF vs. control. The heat map shows the mean log2-fold changes of genes important for osteoblast differentiation from 3 independent experiments at time points 0, 3, 7, 14, and 21 days with a mean exposure of 3 technical replicates each.

**Additional file 2: Fig. S2.** A low frequency alternating magnetic field with an continuous sinusoidal form and very low harmonics (< 1%) with Brms = 5 mT induces an electric potential with Urms = 700 mV in a secondary coil (transducer). The same technique is used in the clinical application”.

**Additional file 3: Fig. S3.** Set up exposure: **A**: 24 wellplates for EF- and combined MF–stimulation. Shown is a plate with gold electrode arrangement. **B**: Primary coil in cooling enclosure for application of the magnetic field. **C**: Tray for 24-well plates with transducer and contacts for electric stimulation. **D**: exposure system within the incubator

**Additional file 4:.** RPKMmean_GOI.xls

## Data Availability

The datasets generated and/or analysed during the current study are available in the supplementary data file: RPKMmean_GOI.xls.
