# Supplementary figures and images for: Exposure of primary osteoblasts to combined magnetic and electric fields induced spatiotemporal endochondral ossification characteristic gene- and protein expression profiles
Source: J Exp Orthop. 2022 May 2;9:39. doi: 10.1186/s40634-022-00477-9 (PMC9061914; doi:10.1186/s40634-022-00477-9)

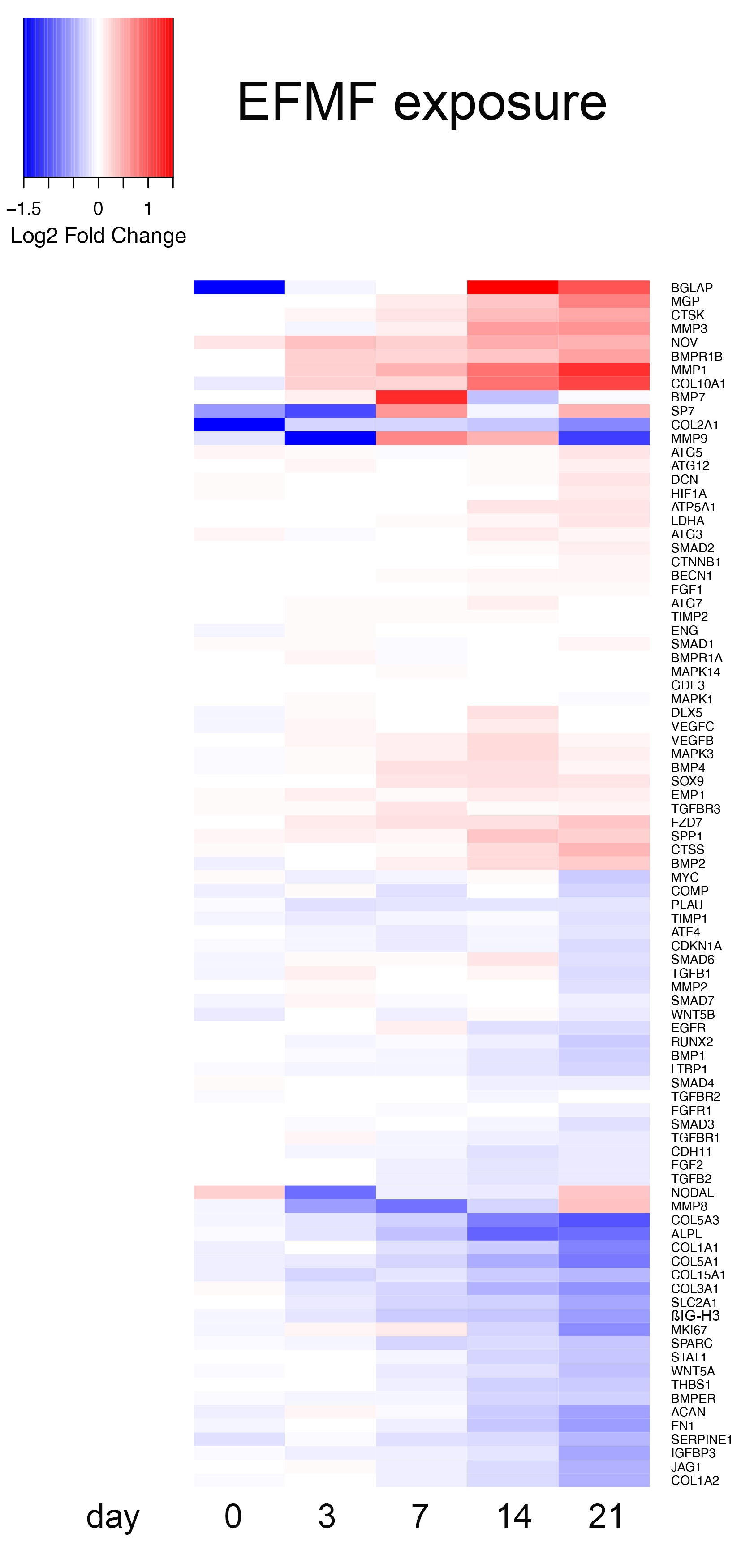

Supplement: Supplementary file 1 — Additional file 1: Fig. S1. Differential expressed genes of human primary osteoblasts treated with EFMF vs. control. The heat map shows the mean log2-fold changes of genes important for osteoblast differentiation from 3 independent experiments at time points 0, 3, 7, 14, and 21 days with a mean exposure of 3 technical replicates each. [file 40634_2022_477_MOESM1_ESM.tif]

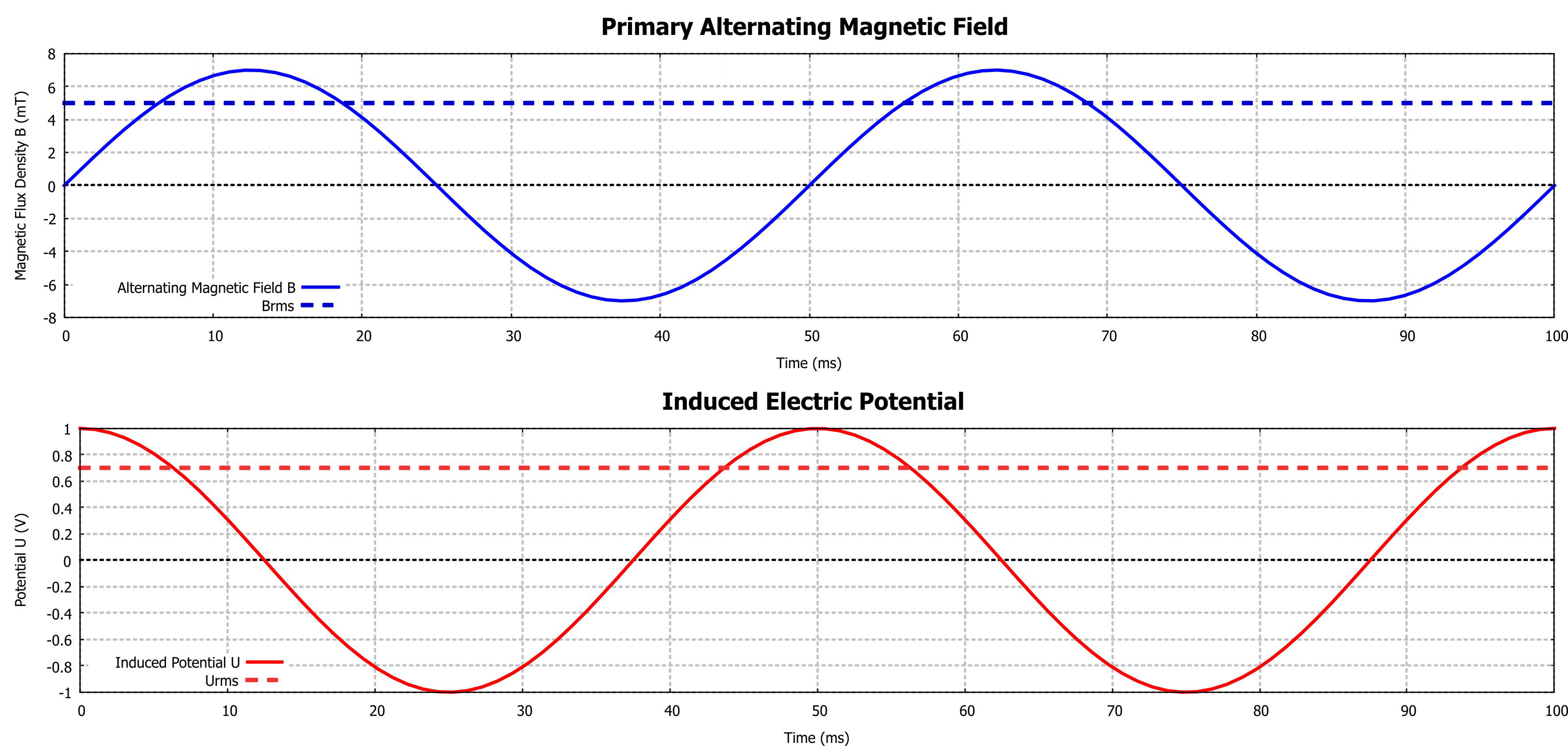

Supplement: Supplementary file 2 — Additional file 2: Fig. S2. A low frequency alternating magnetic field with an continuous sinusoidal form and very low harmonics (< 1%) with Brms = 5 mT induces an electric potential with Urms = 700 mV in a secondary coil (transducer). The same technique is used in the clinical application”. [file 40634_2022_477_MOESM2_ESM.jpg]

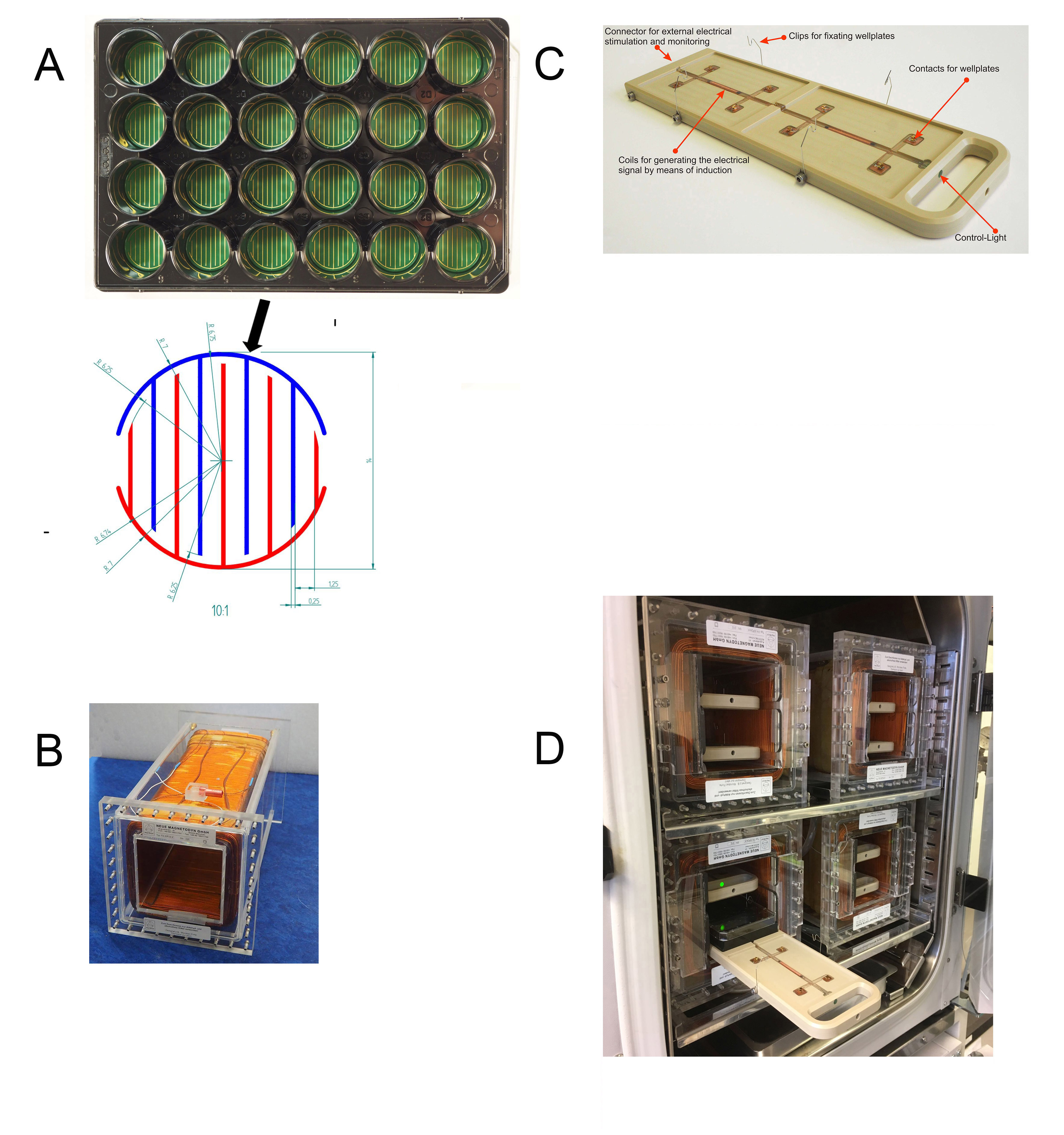

Supplement: Supplementary file 3 — Additional file 3: Fig. S3. Set up exposure: A: 24 wellplates for EF- and combined MF–stimulation. Shown is a plate with gold electrode arrangement. B: Primary coil in cooling enclosure for application of the magnetic field. C: Tray for 24-well plates with transducer and contacts for electric stimulation. D: exposure system within the incubator [file 40634_2022_477_MOESM3_ESM.jpg]
